# Can inflammatory indices predict sentinel lymph node status in patients with early-stage breast cancer?

**DOI:** 10.1097/MD.0000000000034808

**Published:** 2023-08-18

**Authors:** Hakan Balbaloglu, Ilhan Tasdoven, Guldeniz Karadeniz Cakmak

**Affiliations:** a Bulent Ecevit University, School of Medicine, Department of General Surgery, Zonguldak, Turkey.

**Keywords:** axillary lymph node, breast cancer, sentinel lymph node biopsy, systemic immune inflammation index

## Abstract

Breast cancer research has focused on the early detection and treatment of breast cancer. Axillary lymph node status is essential for primary breast cancer staging, recurrence, and survival. The current quest for precision medicine is to identify predictive markers that offer the advantage of individualized treatment options. This study aimed to investigate the value of inflammatory indices in predicting positive sentinel nodes in breast cancer. We studied 602 patients with early-stage breast cancer who underwent sentinel lymph node biopsies (SLNB) at the Bülent Ecevit University General Surgery Clinic. We obtained data, including the clinical and demographic characteristics of the patients, such as age, histological type, and sentinel lymph nodes. Neutrophil, lymphocyte, platelet, and monocyte counts were obtained from preoperative complete blood count test data from the patient registry. The neutrophil-to-lymphocyte ratio (NLR), platelet-to-lymphocyte ratio (PLR), lymphocyte-to-monocyte ratio (LMR), systemic inflammatory index (SII), and sentinel lymph node biopsy were analyzed. Sentinel LAP was negative in 391 (65%) patients and positive in 211 (35%). In the receiver operating characteristic curve analysis, no significant difference was found between SLNB positivity and negativity in terms of NLR, PLR, LMR, or SII. In contrast to previous research, NLR, PLR, LMR, or SII did not affect SLNB positivity prediction in our study.

## 1. Introduction

In a study conducted by the International Agency for Research on Cancer, 2.089 million new cases of breast cancer were detected worldwide in 2018, and 627,000 of these patients died.^[1]^ The number of new breast cancer cases has steadily increased over the last 4 decades, and this trend is expected to continue.^[2–4]^ For this reason, breast cancer research has focused specifically on the early detection and treatment of breast cancer. Complete surgical success in breast lesions requires precise localization and accurate assessment of the lymph node status. Numerous studies have been conducted in these areas. Ultrasound or mammography-guided wire localization allows for immediate localization before surgery, reducing hospitalization time and healthcare costs.^[5]^ Moreover, the modified Sentinel Node and Occult Lesion Localization technique has demonstrated an excellent surgical diagnostic rate for both non-palpable lesions and the sentinel lymph node.^[6]^ Axillary lymph node (ALN) status is essential for staging, recurrence, and survival of patients with primary breast cancer.^[7]^ ALN values were evaluated using axillary lymph node dissection (ALND) or sentinel lymph node biopsy (SLNB).^[8,9]^ Both interventions are invasive and associated with specific morbidities, such as pain, lymphedema, numbness, tingling sensation, and impaired arm use.^[10]^ Fortunately, SLNB has replaced ALND in patients with clinically node-negative breast cancer with evidence-based medicine, confirming the oncologic safety of SLNB.^[11,12]^ Therefore, the preoperative nodal evaluation of early-stage breast cancer is critical.^[13]^ The current quest for precision medicine is to find predictive markers that offer the advantages of individualized treatment options for each case. The effects of inflammation on all stages of tumorigenesis playing crucial roles in cancer prognosis, have been demonstrated in previous studies.^[14]^ From this perspective, the ratios of inflammatory cells, including the neutrophil-to-lymphocyte ratio (NLR), platelet-to-lymphocyte ratio (PLR), and lymphocyte-to-monocyte ratio (LMR), have been used to evaluate the inflammatory response, which could affect the prognosis of various types of cancer.^[15–19]^ The systemic immune inflammation index (SII = N × P/L) is a new marker of inflammation based on platelet (P), neutrophil (N), and lymphocyte (L) ratios^[20]^ and has been suggested to predict prognosis in cancer patients.^[21,22]^

This study investigated the value of peripheral blood NLR, PLR, LMR, and systemic immune inflammation index in predicting positive sentinel nodes in breast cancer cases.

## 2. Materials and methods

The records of patients who underwent SLNB with early-stage breast tumors were retrospectively reviewed between January 2013 and April 2021 were retrospectively reviewed. The inclusion criteria were female patients with invasive ductal carcinoma, stage cT1-2N0M0 solitary breast tumors, SLNB, and accessible patient records. The study sample excluded cases with clinical evidence of infection, cardiac or autoimmune and inflammatory rheumatic diseases, hematological system diseases, preoperative chemoradiotherapy, steroid use, nonsteroidal anti-inflammatory drug use, or incomplete medical records. As a result of the evaluation of the patient records, 602 patients were included in the study. Data, including the clinical and demographic characteristics of the patients, such as age, histological type of breast cancer, sentinel lymph node status, and preoperative complete blood count (CBC) results, were obtained from the patient registry system of Bulent Ecevit University (MIA Med.).

### 2.1. Hematological analyses

An automated hematology analyzer (UniCel DxH 800, BECKMAN COULTER, USA) was used for the CBC tests.

Neutrophil (N), lymphocyte (L), platelet (P), and monocyte (M) counts were obtained based on the preoperative CBC test data from the patient registry. The NLR and PLR values were calculated by dividing the neutrophil and platelet counts by the absolute lymphocyte count. The LMR was calculated by dividing the lymphocyte count by the absolute monocyte count. The systemic inflammatory index was obtained by multiplying the neutrophil count by PLR, as described by Feng et al^[20]^

### 2.2. Statistical analyses

Statistical analyses were performed using the Statistical Package for the Social Sciences (SPSS Inc., Chicago, IL) 22-package program. In the results, as descriptive statistics, n and % values were used to present categorical data, and mean ± standard deviation (Mean ± SD) and median (minimum-maximum) values were used to present continuous data. Chi-square analysis (Pearson chi-squared test) was performed to compare categorical variables between the groups. The Kolmogorov-Smirnov test was used to evaluate the normality of the distributions of the continuous variables. The Mann–Whitney *U* test was used to compare 2 independent groups of non-normally distributed data. Receiver operating characteristic curves were drawn to measure the values of NLR, PLR, LMR, and SII for predicting LAP positivity. Spearman correlation analysis was performed to determine the relationships between the measured variables. The level of statistical significance was set at *P* < .05.

## 3. Results

**Figure 1.**
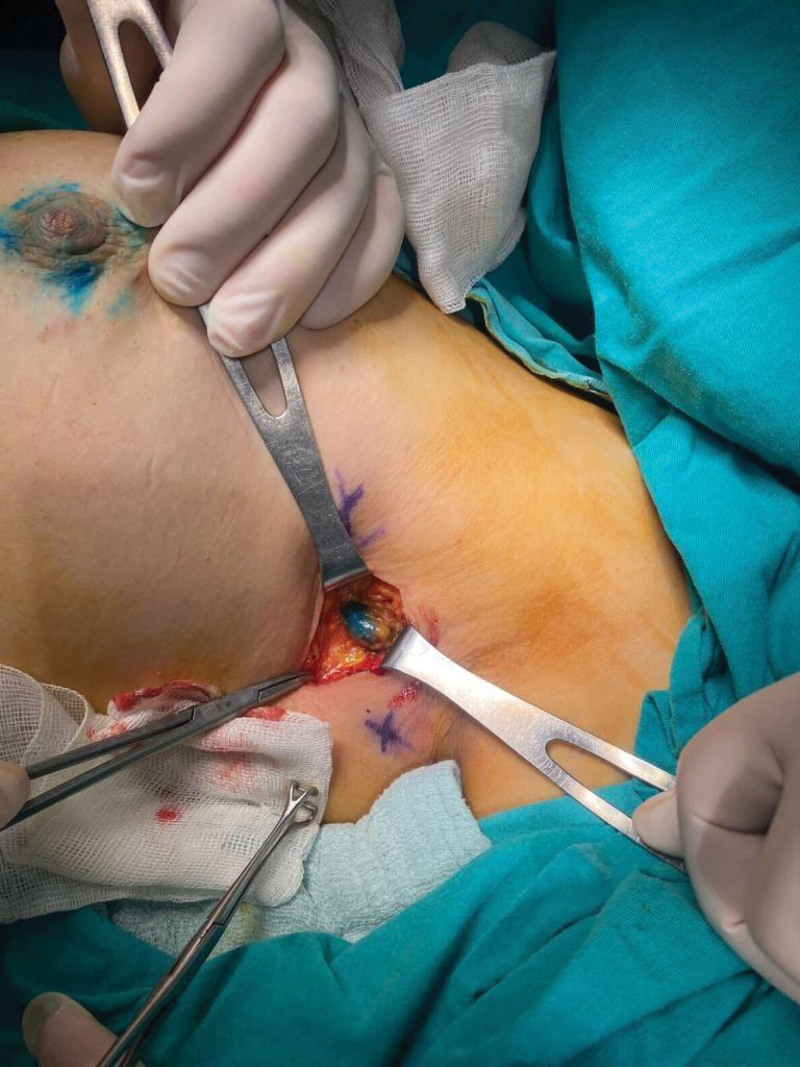
Early-stage breast cancer patients who underwent sentinel lymph node dissections at our clinic.

**Figure 2.**
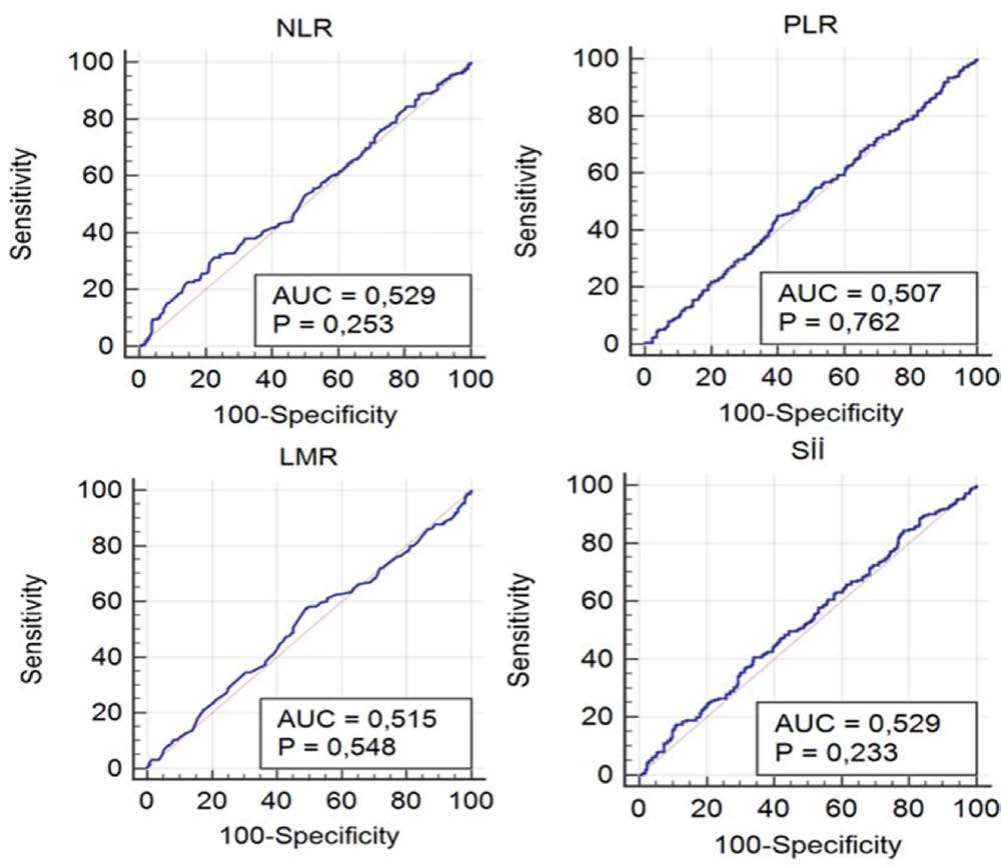
ROC curves of NLR, PLR, LMR, and SII values for LAP positivity. LMR = lymphocyte-to-monocyte ratio, NLR = neutrophil-to-lymphocyte ratio, PLR = platelet-to-lymphocyte ratio, ROC = receiver operating characteristic, SII = systemic inflammatory index.

The sample included 602 patients with early-stage breast cancer who underwent sentinel lymph node dissection at our clinic (Fig. 1). The mean age of the patients was 53.56 ± 11.83 (min = 29–max = 86). Sentinel LAP was negative in 391 (65%) patients and positive in 211 (35%). The mean age of the LAP-negative patients was 53.20 ± 11.94, and that of the LAP-positive patients was 54.23 ± 11.62. There was no significant difference in age between the negative and positive groups (*P* = .323). There was no significant difference between sentinel LAP positivity and negativity in terms of neutrophil count (*P* = .263), lymphocyte count (*P* = .593), platelet count (*P* = .937), monocyte count (*P* = .158), NLR (*P* = .247), PLR (*P* = .762), LMR (*P* = .541), or SII (*P* = .233) values (Table 1). Receiver operating characteristic analysis was conducted to investigate the capacity of the examined ratios to predict SLAP positivity, and the cutoff values were determined. With a cutoff value of 1.52 NLR, a sensitivity of 30.81% and a specificity of 77.75% were found, and it was determined that NLR had no significant diagnostic value in terms of LAP positivity (*P* = .253). With a cutoff value of 114 for PLR, a sensitivity of 45.02% and a specificity of 59.85% were found, and PLR had no significant diagnostic value in terms of LAP positivity (*P* = .762). With a cutoff value of 3.88 for LMR, a sensitivity of 57.35% and specificity of 51.15% were found, and it was determined that LMR had no significant diagnostic value in terms of LAP positivity (*P* = .548). With a cutoff value of 428.56 for SII, a sensitivity of 40.76% and a specificity of 65.98% were found, and it was determined that the SII had no significant diagnostic value in terms of LAP positivity (*P* = .233) (Table 2, Fig. 2).

**Table 1:** Comparison of characteristics of patients according to sentinel LAP status.

	Negative (n = 391)	Positive (n = 211)	Total (n = 602)	*P* value*
	Mean ± standard deviation Median (min-max)	Mean ± standard deviation median (min-max)	Mean ± standard deviation Median (min-max)	
Age	53.20 ± 11.94 53.00 (29.00–86.00)	54.23 ± 11.62 54.00 (30.00–83.00)	53.56 ± 11.83 54.00 (29.00–86.00)	.323
Neutrophil	4.50 ± 1.86 4.10 (.20–17.00)	4.33 ± 1.77 3.90 (.50–12.20)	4.44 ± 1.83 4.10 (.20–17.00)	.263
Lymphocyte	0.53 ± 0.19 0.50 (0.10–1.50)	0.54 ± 0.32 0.50 (0.10–4.00)	0.54 ± 0.24 0.50 (0.10–4.00)	.593
Platelet	263.55 ± 77.13 251.00 (83.00–718.00)	258.86 ± 68.13 260.00 (65.00–528.00)	261.90 ± 74.07 254.50 (65.00–718.00)	.937
Monocyte	0.53 ± 0.21 0.50 (0.00–2.30)	0.55 ± 0.21 0.50 (0.10–1.90)	0.53 ± 0.21 0.50 (0.00–2.30)	.158
NLR	2.56 ± 2.29 2.04 (0.25–31.00)	2.74 ± 4.56 2.00 (0.71–61.00)	2.62 ± 3.27 2.00 (0.25–61.00)	.247
PLR	147.40 ± 97.37 127.37 (41.36–1415.00)	152.16 ± 161.37 122.50 (39.50–2240.00)	149.06 ± 123.52 124.31 (39.50–2240.00)	.762
LMR	4.22 ± 1.83 3.83 (0.50–17.00)	4.33 ± 2.10 4.13 (0.10–18.00)	4.26 ± 1.93 4.00 (0.10–18.00)	.541
SII	682.44 ± 688.48 523.88 (33.25–8773.00)	690.98 ± 1072.45 504.58 (137.65–13664.00)	685.43 ± 842.31 515.91 (33.25–13664.00)	.233

LMR = lymphocyte-to monocyte ratio, NLR = neutrophil-to lymphocyte ratio, PLR = platelet-to lymphocyte ratio, SII = systemic immune inflammation index.

* Mann–Whitney *U* test. Statistical significance = *P* < .05.

**Table 2:** Specificity and sensitivity of the measured parameters in determining LAP positivity.

	AUC	*P* value			Sensitivity	Specificity	PPV	NPV
			Lower	Upper				
NLR ≤ 1,52	0.529	.253	0.488	0.569	30.81	77.75	42.8	67.6
PLR ≤ 114	0.507	.762	0.467	0.548	45.02	59.85	37.7	66.9
LMR > 3,88	0.515	.548	0.474	0.556	57.35	51.15	38.8	69.0
SII ≤ 428,56	0.529	.233	0.489	0.570	40.76	65.98	39.3	67.4

AUC = area under ROC curve, LMR = lymphocyte-to monocyte ratio, NLR = neutrophil-to lymphocyte ratio, NPV = negative predictive value, PLR = platelet-to lymphocyte ratio, PPV = positive predictive value, SII = systemic immune inflammation index.

## 4. Discussion

This study investigated whether NLR, PLR, LMR, and SII are potential markers for predicting sentinel lymph node (SLN) positivity in early-stage breast cancer. To the best of our knowledge, no study has examined the combined role of NLR, PLR, LMR, and SII in evaluating SLN positivity in early-stage breast cancer. The lymph node that is affected first during tumor spread is called the sentinel lymph node, and biopsy of this lymph node has become the standard method for early breast cancer staging.^[23]^ Accurate ALN status detection is critical for both the survival prediction and treatment of breast cancer.^[24]^ However, the search for alternative methods for evaluating lymph node metastasis continues. Tumor progression is a complex condition that results from interactions between tumor cells and healthy cells.^[25]^ A part of the host response is the nonspecific systemic inflammatory response, which includes the release of pro-inflammatory cytokines and growth factors that can promote growth in some tumors, thus affecting survival.^[26]^ Inflammatory processes are thought to lead to angiogenesis, invasion, and metastasis of cells in the tumor area.^[27]^ Neutrophils promote the adhesion of circulating tumor cells to distant sites and induce tumor growth.^[28]^ Platelets play a role in cancer progression by facilitating adhesion of tumor cells to the endothelium and preventing cell death and distant metastasis.^[29]^ Lymphocytes play an active role in the immune response against tumor cells, reduce metastasis, inhibit the proliferation of tumor cells, and induce cytotoxic cell death.^[30]^ From this point of view, it can be thought that PLR, NLR, LMR, and SII have predictive capacity for the prognosis of breast cancer. Correlations between SII, NLR, LMR, PLR, and prognosis in various types of cancer have been revealed.^[31–35]^ In a meta-analysis of 16 studies involving 4875 patients that evaluated the association of SII with solid tumor prognosis, Zhong et al^[36]^ found a significant relationship between high SII and poor survival. According to Sokmen et al^[37]^, a low LMR and high NLR, PLR, and SII suggest the possibility of advanced breast cancer. Koh et al^[38]^ determined that higher NLR and PLR values are independently associated with a higher risk of mortality due to breast cancer. Morkavuk et al^[39]^ reported that the success rate of PLR in the diagnosis of metastatic SLN in early-stage breast cancer was higher than that of ultrasound and other imaging methods. Atak et al stated that the preoperative NLR is a valuable diagnostic tool for predicting non-SLN metastasis.^[40]^ Ma et al^[41]^ argued that it might be appropriate to use LMR as a prognostic marker in patients with breast cancer, and LMR is a better prognostic marker than NLR and PLR in predicting breast cancer prognosis.

Contrary to the studies mentioned above, there was no significant difference between SLNB positivity and negativity in terms of NLR, PLR, LMR, or SII in our study. Yang et al^[42]^ did not find a significant relationship between NLR and SLN metastasis status in patients, similar to our study, but they considered a high PLR and vascular tumor thrombi as risk factors for SLN metastasis. In contrast, Laohawiriyakamol et al^[43]^ reported that NLR is a useful tool for predicting non-sentinel lymph node metastasis in breast cancer patients. Based on the relationship between lymph node involvement and prognosis,^[44]^ we believe that there is substantial confusion about whether inflammatory indices are effective markers for determining the prognosis of early-stage breast cancer.

The limitations of our study include its retrospective design. The counts of inflammatory cells in the peripheral blood can be affected by many independent factors, such as the patient’s physical condition and diet, and this study reflects a single-center experience.

## 5. Conclusion

Evolutionary changes and minimally invasive interventions have come to the forefront in the diagnosis and treatment of many diseases. Evaluation of ALN involvement without ALND or SLNB in early-stage breast cancer will reduce morbidity rates and facilitate treatment planning. In this study, we aimed to identify an auxiliary marker in addition to existing tests showing ALN involvement in patients with early-stage breast cancer.

We investigated whether inflammatory indices can be used as adjunctive markers to assess the status of SLN in early-stage breast cancer. Contrary to previous studies in the literature, NLR, PLR, LMR, or SII did not affect the prediction of LAP positivity in our study. Prospective and multicenter studies are required to investigate this topic comprehensively.

## Author contributions

**Conceptualization:** Hakan Balbaloglu.

**Data curation:** Hakan Balbaloglu, Ilhan Tasdoven, Guldeniz karadeniz Cakmak.

**Formal analysis:** Guldeniz karadeniz Cakmak.

**Funding acquisition:** Ilhan Tasdoven, Guldeniz karadeniz Cakmak.

**Investigation:** Ilhan Tasdoven, Guldeniz karadeniz Cakmak.

**Methodology:** Hakan Balbaloglu, Ilhan Tasdoven, Guldeniz karadeniz Cakmak.

**Project administration:** Hakan Balbaloglu, Guldeniz karadeniz Cakmak.

**Resources:** Hakan Balbaloglu, Guldeniz karadeniz Cakmak.

**Software:** Hakan Balbaloglu, Ilhan Tasdoven, Guldeniz karadeniz Cakmak.

**Supervision:** Hakan Balbaloglu.

**Validation:** Hakan Balbaloglu.

**Visualization:** Hakan Balbaloglu, Ilhan Tasdoven, Guldeniz karadeniz Cakmak.

**Writing – original draft:** Hakan Balbaloglu.

**Writing – review & editing:** Hakan Balbaloglu, Guldeniz karadeniz Cakmak.
